# A Proposed Methodology to Deal with the Impact of In Vitro Cellular Matrix on the Analytical Performances of a Targeted Metabolomic LC-HRMS Method

**DOI:** 10.3390/ijms24043770

**Published:** 2023-02-13

**Authors:** Jérôme Guitton, Floriane Gavotto, Emeline Cros-Perrial, Lars Petter Jordheim, Christelle Machon

**Affiliations:** 1 Biochemistry and Toxicology Laboratory, Lyon Sud Hospital, University Hospital of Lyon, CEDEX, 69495 Pierre-Bénite, France; 2 Inserm U1052, CNRS UMR5286 Centre de Recherche en Cancérologie de Lyon, 69000 Lyon, France; 3 Toxicology Laboratory, Faculty of Pharmacy, University of Lyon, 69008 Lyon, France; 4 Luxembourg Centre for Systems Biomedicine, University of Luxembourg, L-4367 Belvaux, Luxembourg; 5 Analytical Chemistry Laboratory, Faculty of Pharmacy, University of Lyon, 69008 Lyon, France

**Keywords:** targeted metabolomic, liquid chromatography, high resolution mass spectrometry, cell extracts, analytical performances, matrix effect

## Abstract

Performances of metabolomic methods have been widely studied on biological matrices such as serum, plasma, and urine; but much less on in vitro cell extracts. While the impact of cell culture and sample preparation on results are well-described, the specific effect of the in vitro cellular matrix on the analytical performance remains uncertain. The aim of the present work was to study the impact of this matrix on the analytical performance of an LC-HRMS metabolomic method. For this purpose, experiments were performed on total extracts from two cell lines (MDA-MB-231 and HepaRG) using different cell numbers. Matrix effects, carryover, linearity, and variability of the method were studied. Results showed that the performances of the method depend on the nature of the endogenous metabolite, the cell number, and the nature of the cell line. These three parameters should, therefore, be considered for the processing of experiments and the interpretation of results depending on whether the study focuses on a limited number of metabolites or aims to establish a metabolic signature.

## 1. Introduction

Nuclear magnetic resonance (NMR) and mass spectrometry (MS) are the two most commonly used modes of detection in metabolomics. Although NMR presents many advantages, such as being a fast and non-destructive method, its relatively low sensitivity limits the analysis of low-abundance metabolites [1]. Due to its higher sensitivity, MS is the detection method of choice for metabolomics in biological matrices [1]. MS is coupled with a separative method such as gas chromatography, capillary electrophoresis, or liquid chromatography (LC), the latter being the most frequently used for untargeted and targeted metabolomic analysis [1]. The use of LC coupled with high-resolution mass spectrometry (HRMS) methods allows higher selectivity, sensitivity, accuracy, and precision [1]. However, ion suppression and low ionization efficiency can limit the application of LC-MS with complex matrices. These drawbacks are well-known since many applications in the field of health sciences have described metabolomic approaches from complex biological matrices such as blood, urine, tissue, cerebrospinal fluid, etc. [1]. Although several authors applied LC-MS metabolomics methods on in vitro cell extracts [2,3,4,5,6,7,8,9,10], data on the methodology used to evaluate analytical performances considering the cellular matrix remain only scarcely detailed. Serafimov et al. used standard addition experiments to evaluate the performances of an LC-MS/MS method [6], whereas other works performed method evaluation on a surrogate matrix or water [4,5].

Hounoum et al. found that for extracts from a given cell line, there is a biological variability depending on parameters such as the culture medium, the cellular density, the proliferation cellular rate, etc. [11]. The direct consequence is variability in the number of endogenous metabolites from one culture batch to another. Furthermore, as shown in previous works conducted on in vitro cell extracts, the number and the abundance of metabolites extracted vary according to several pre-analytical parameters, including the detachment of adherent cells by scraping or trypsinization, nature, and volume of the lysis solvent and the duration of contact with the lysis solvent [6,7,12]. However, the specific impact of the in vitro cellular matrix on analytical performances has been barely reported. Indeed, the matrix effect (ME) related to in vitro cell extracts has only been studied in two papers, and the values reported depend on the approach used to evaluate the ME. While Serafimov et al. showed an important ME for some metabolites (up to 42%) based on a standard addition approach, Zhu et al. observed a ME of less than 20% using a surrogate matrix approach [4,6]. Despite these data, the knowledge about the specific impact of the in vitro cellular matrix on the analytical performances of LC-MS/MS methods appears to be insufficient, and several questions remain. These concern the protocols for the evaluation of analytical performances for in vitro cell extracts, the impact of the cell number on analytical performances, and whether or not the analytical performances are similar between different cell lines.

In the present work, based on an LC-HRMS analysis, we have demonstrated the impact of the nature of the cell line and the cell number on analytical performances (linearity, ME, variability). Based on these results, keys for workflow analysis and data processing in a quantitative approach from in vitro cell extracts are proposed.

## 2. Results

### 2.1. LC-HRMS Method

The reversed-phase chromatographic method adapted from Liu et al. was optimized on 88 metabolites using pure standards (Table S1) [13]. These metabolites belong principally to amino acids, tricarboxylic acids, polyamines, phosphorylated hexose, nicotinamide, and nucleosides. The chromatographic challenge was to obtain sufficient retention for polar metabolites, such as some amino acids and polyamines, and to separate isomers of leucine. The column breakthrough has also been considered during the selection of the column and the mobile phases. Finally, a reversed-phase column with a polar endcapping was preferred to HILIC columns mainly because of an insufficient resolution between isomers with HILIC. The lowest retention time (RT) was 2.1 min for spermine, with a dead time estimated at 1.8 min. The highest was 17.8 min for methylhippuric acid. The three isomers of leucine (same exact mass and fragmentation spectra) were well-separated (resolution factor superior to 1.5) with RT of 9.3, 9.7, and 10.1 min for norleucine, isoleucine, and leucine, respectively.

Metabolites were identified by their RT, the exact mass of the parent ion coupled to the isotopic pattern (acquired in the full scan mode), and the exact mass of fragments coupled to the fragmentation spectrum (acquired with the ddMS2 mode). The use of an HRMS allows a high selectivity. Therefore, by using 5 ppm as mass precision, co-eluted metabolites with a mass shift equal to 1 did not interfere with the isotopic pattern. For example, the m+1 isotope of asparagine ([M+H]^+^ = 134.06412) and the molecular ion of aspartic acid ([M+H]^+^ = 134.04478), or the m+1 isotope of glutamine ([M+H]^+^ = 148.07977) and the molecular ion of glutamic acid ([M+H]^+^ = 148.06040), were all selectively identified. Quantification was performed using the m/z of the parent ion obtained with the full scan mode (Table S1).

### 2.2. Performances of the Method

After optimization of the separation and the identification of metabolites, the performances of the LC-HRMS method were evaluated on cell extracts from MDA-MB-231 and HepaRG cell lines. All 88 metabolites were monitored. At 7 × 10^5^ cells, sixty-three (71.6%) and sixty-four (72.7%) metabolites were identified in MDA-MB-231 and HepaRG cell extracts, respectively, with sixty-two in common for both cell lines.

The robustness of the LC method was confirmed by the study of the variability of RT for each metabolite. After analysis of 30 samples of each cell line, the variability was lower than 5% for all metabolites, except for phosphoglyceric acid (CV = 8.4%) in MDA-MB-231 cells and oxidized glutathione (CV = 6.6%) in HepaRG extracts. A carryover was observed for four metabolites for MDA-MB-231 extracts and eight metabolites for HepaRG extracts. However, since the highest value was 2.8% (for phenylalanine in HepaRG cells), the carryover was considered as not significant.

**Figure 1:**
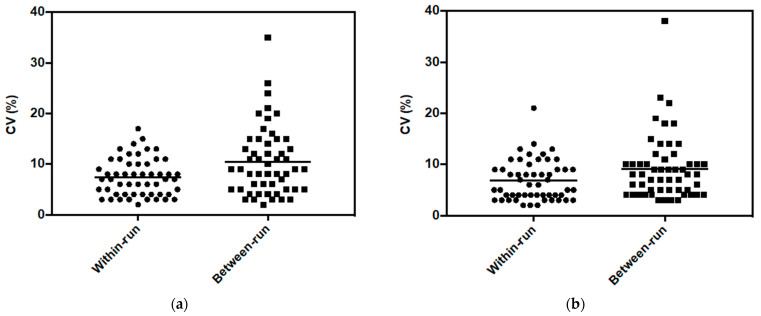
Distribution of within-run and between-run coefficient of variation of peak area for metabolites in (**a**) MDA-MB-231 cells and (**b**) HepaRG cells, from 2 × 10^5^ cells.

The within-run and the between-run variabilities of the peak area were compared for each metabolite and each cell line (Figure 1). The average and dispersion of between-run values were higher than those of within-run for both cell lines. In the following experiments, data were obtained by carrying out the analyses within the same series; therefore, only the within-run variability was considered.

**Figure 2:**
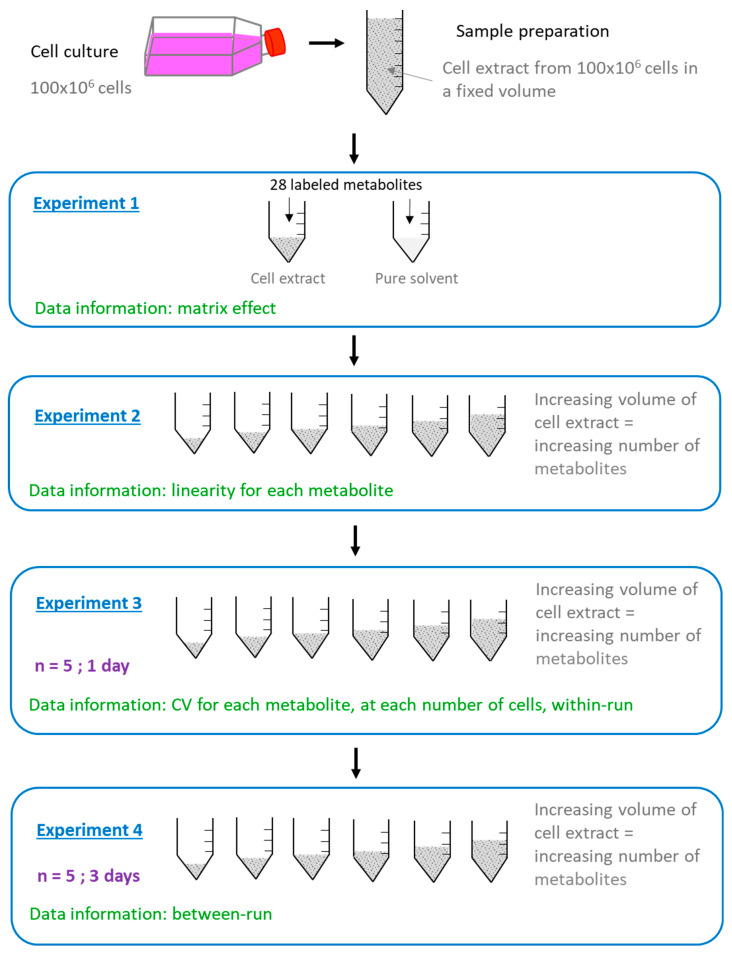
Workflow applied for the study of the impact of in vitro cell extracts on the performances of the LC-HRMS method. Each experiment was performed on MDA-MB-231 and HepaRG cell extracts.

We further applied the following workflow in order to study the influence of the in vitro cell extracts on the performances of the analytical method (Figure 2).

#### 2.2.1. Matrix Effect

**Figure 3:**
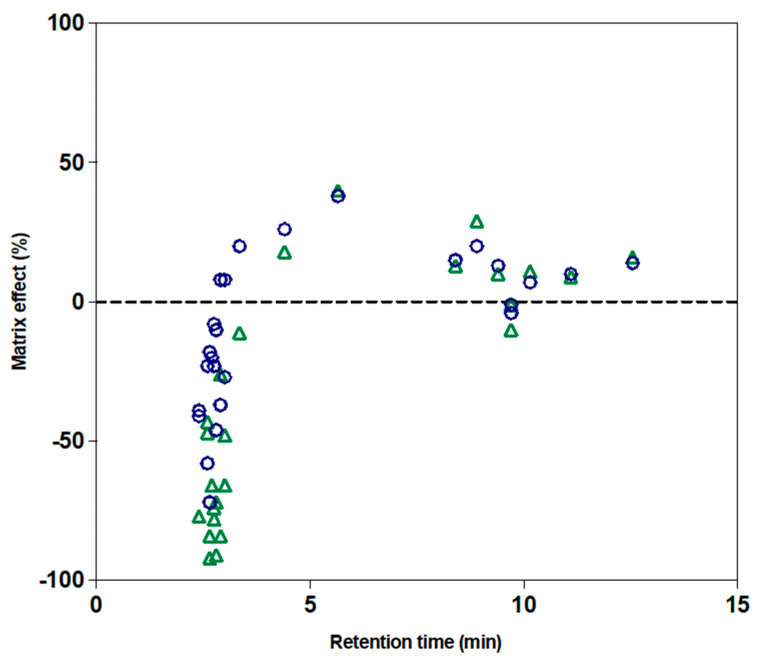
The matrix effect (ME) of the 28 labeled metabolites as a function of retention time in MBAMB-231 cell extracts. Blue circles: the ME for a cell extract corresponding to 1 × 10^5^ cells. Green triangles: the ME for a cell extract corresponding to 5 × 10^5^ cells.

Blank cell extracts were not available since the compounds studied were endogenous metabolites. Thus, the ME was determined using an approach with surrogate analytes of endogenous metabolites, including 28 labeled metabolites spiked at the same level in cell extracts and in a pure solution of the mobile phase. The ME ranged from −92% to +40%. The RT appeared as an important parameter since analytes eluted before 3 min were associated with higher negative values of the ME (Figure 3). This could be explained by an alteration of the ionization due to competition between a high number of compounds in the ion source. For the short RTs, the MEs were also increased together with the cell number used for the extraction (1 × 10^5^ vs. 5 × 10^5^ cells, Figure 3).

**Figure 4:**
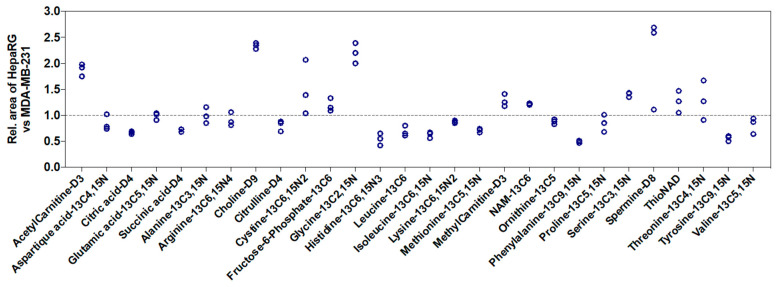
Relative response of the 28 labeled metabolites spiked in cell extracts from HepaRG cells compared with MDA-MB-231 cells (*n* = 3, at 3 × 10^5^ cells for all experiments). Peak areas in MDA-MB-231 cells were considered as the reference and normalized to 1. Rel.: relative.

We also studied the influence of the nature of cell lines on signal intensities. Using the peak area of 28 labeled metabolites spiked in MDA-MB-231 cell extracts as a reference, we observed an increase in peak area up to 2.5-fold or a decrease up to 2-fold in HepaRG cell extracts (Figure 4). Thus, this showed that the ME from in vitro cell extracts depends on the metabolite, the cell number, and the nature of cell lines.

#### 2.2.2. Linearity

**Figure 5:**
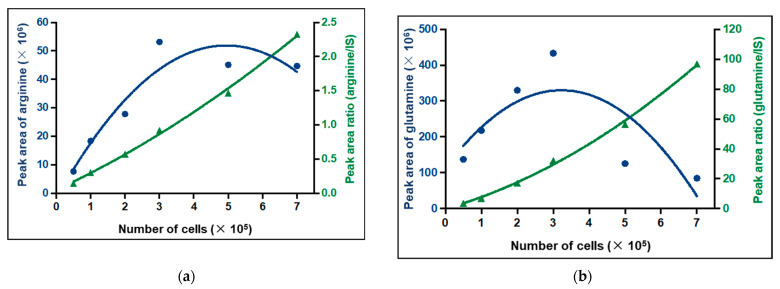
Calibration curves of (**a**) arginine and (**b**) glutamine by external calibration (blue) and internal calibration (green). IS: internal standard.

The linearity of the method was assessed by the comparison between calibration curves established by external and internal calibration. The *Y*-axis corresponded to the area of the chromatographic peak of the metabolite or the peak area ratio (peak area of metabolite/peak area of internal standard) and the *X*-axis corresponded to the cell number used. All calibration curves were polynomial. Due to the ME which depends on the cell number for some metabolites, the area of the chromatographic peak was not always correlated with the cell number. We even observed a decrease in the area of the chromatographic peak when the cell number increased for some metabolites with RT lower than 3 min, such as arginine and glutamine (Figure 5). Thus, internal calibration with an internal standard that corrected the ME was selected for all metabolites. That is why 28 internal standards (IS) were used and the choice of IS for each metabolite was based on the chemical similarity, the RT, the ionization mode, and the ME (Table S1). Thus, the results were expressed as the peak area ratio (peak area of metabolites/peak area of IS) (Figure 5, green lines).

**Figure 6:**
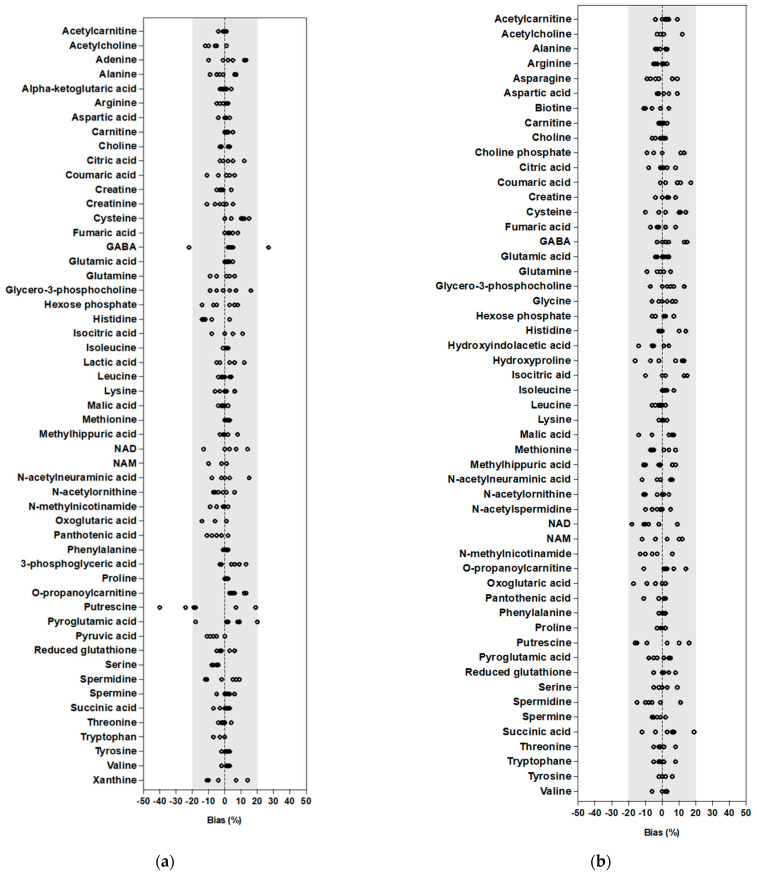
The bias of linearity assessment in (**a**) MDA-MB-231 and (**b**) HepaRG cell extracts during method validation. Results are expressed as bias between the calculated concentration and the theoretical concentration. The grey area corresponds to the acceptance criteria (±20%).

Having determined and chosen the internal calibration mode, the linearity of the method was assessed in both cell lines. Calibration experiments were performed with cell extracts from 0.5 × 10^5^ to 7 × 10^5^ cells. Curves were established and performances were calculated only for metabolites showing a signal from 0.5 × 10^5^ or 1 × 10^5^ to 7 × 10^5^ cells (corresponding to at least five standards). This represented a total of 53 metabolites, among which 49 were common to both cell lines. Linearity, based on bias between theoretical and calculated cell number, was validated (<20%) for all metabolites, except GABA and putrescine for MDA-MB-231 cells (Figure 6). To be noted, the linearity of these two metabolites was validated in HepaRG cells, indicating an impact of the nature of cell line also on linearity.

### 2.3. Impact of the Nature of the Cell Line and the Cell Number on Analytical Performances

We next evaluated the impact of the nature of the cell line and the cell number on analytical performances by studying the method variability. The coefficient of variation (CV) of each metabolite was calculated based on the results of five replicates for 0.5, 1, 2, 3, 5, and 7 × 10^5^ cells for each cell line.

**Figure 7:**
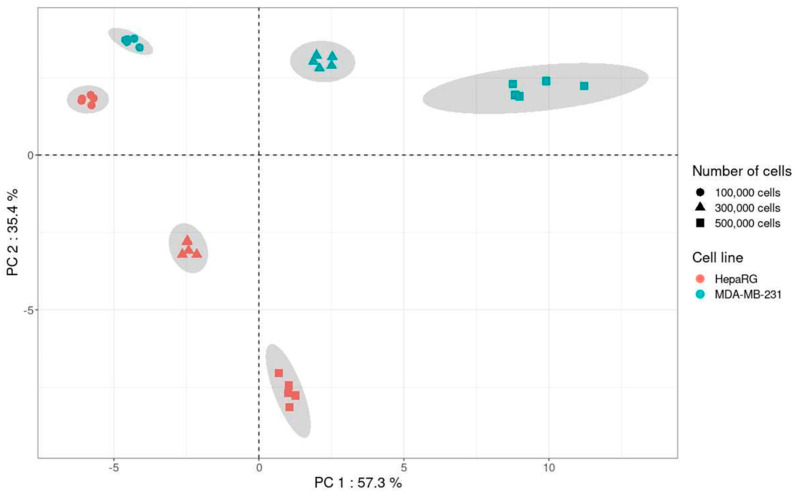
Principal component analysis including the metabolites present in MDA-MB-231 and HepaRG cell lines (*n* = 5 for each cell number and each cell line).

A principal component analysis (PCA) including the quantitatively analyzed metabolites in both cell lines was performed using CV values from 1, 3, and 5 × 10^5^ cells for each cell line (Figure 7).

Results of PCA showed a clear impact of the nature of the cell line and the cell number on the variability of the method. Indeed, for the metabolites analyzed with the present method, the variability was lower with 1 × 10^5^ cells than with 3 and 5 × 10^5^ for both cell lines. Thus, in addition to providing information on the impact of the in vitro cell matrix on the variability of the method, PCA allows the selection of the optimal cell number, i.e., 1 × 10^5^ cells.

**Figure 8:**
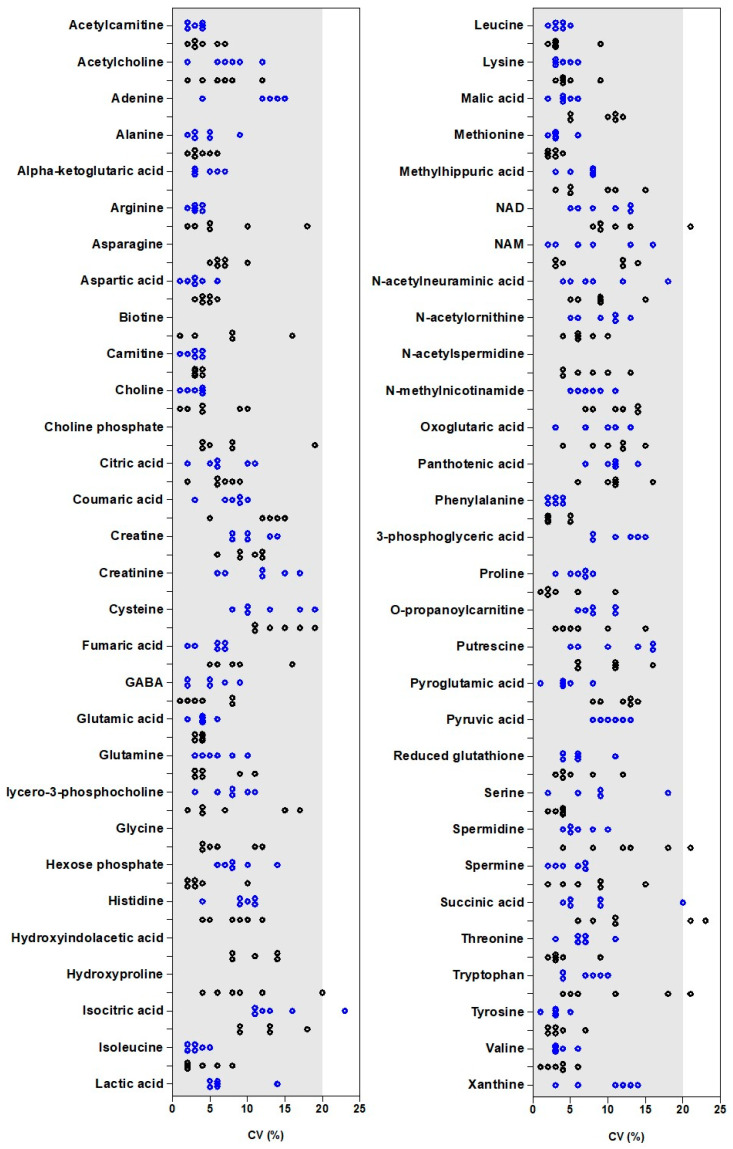
Coefficient of variation (*n* = 5) of each metabolite in each cell line for 0.5, 1, 2, 3, 5, and 7 × 10^5^ cells. Results obtained for MDA-MB-231 (blue) and HepaRG (black) are presented. For some metabolites, the establishment of a calibration curve was possible only in one of the two cell lines (for example, adenine in MDA-MB-231).

However, the PCA approach did not inform on the variability of each metabolite and how the cell line and cell number could impact this. To complete these results, the individual CV of each metabolite for 0.5, 1, 2, 3, 5, and 7 × 10^5^ cells for each cell line are presented (Figure 8).

The detailed study of the variability for each metabolite showed that most CV values were less than 20%, indicating a low variability. Some metabolites exhibited one or two CV values higher than 20%, including isocitric acid (at 5 × 10^5^ cells) in MDA-MB-231, spermidine (at 2 × 10^5^ cells), succinic acid (at 0.5 and 1 × 10^5^ cells), NAD (at 0.5 × 10^5^ cells), and tryptophan (at 0.5 × 10^5^ cells) in HepaRG cells.

## 3. Discussion

Here, using two cell lines (MDA-MB-231 and HepaRG) and different cell numbers, we have studied the impact of the in vitro cellular matrix on the performances of an LC-HRMS metabolomic method. In the following discussion, we propose methods that can be implemented in the experimental layout to handle this.

The first challenge was to evaluate the ME, knowing that an analyte-free biological matrix is not available for cell extracts. The ME is known to be present in metabolomics studies and to differ between metabolites [14]. It can be defined as alterations in the ionization efficiency by the presence of co-eluting molecules [15]. It varies according to the metabolite, the LC method, the ion source, and the matrix itself. Concerning the two chromatographic modes mostly used in metabolomics, namely reversed phase and Hydrophilic interaction chromatography (HILIC), it was reported that there was a higher ME in the second one [16,17]. The comparison of the measured ME from the present work and Serafimov et al. showed similar high values for the ME (from 42 to 110% for Serafimov et al.) [6] while reversed-phase and HILIC were used. Although an ESI source is more prone to the ME than atmospheric pressure chemical ionization (APCI) and atmospheric pressure photoionization (APPI) sources, it remains the largest ion source used in metabolomics [18]. Finally, concerning the matrix itself, Serafimov et al., by using standard addition and the present work by using surrogate analytes, observed a large distribution of the values for the ME (Figure 3). Inversely, Zhu et al., with a spiked surrogate matrix produced by charcoal stripping, reported ME values of less than 20% [4,6]. However, the composition of a surrogate matrix is different than the original one, leading to different behaviors against extraction recovery and the ME [19].

Our results also showed that the ME depends on the nature of the cell line. It may be linked to the intracellular content which differs between cell lines. For example, for the same cell number, HepaRG cells exhibited higher protein content than MDA-MB-231 cells (2.5-fold higher in HepaRG cells). The ME was worsened by an increasing cell. This implies:

- that all metabolomics experiments which aim to compare two or more conditions (i.e., treated vs. non-treated cells, sensitive vs. resistant cells) have to be performed with the same cell number for each sample.
- that comparison of results obtained for a given metabolite between two cell lines should be performed with caution. Either the ME of the metabolite of interest is proven to be identical for the two cell lines and results can be directly compared, or the cell lines present a different ME, and an absolute quantification is needed to compare results.
- the use of a sufficient number of IS in order to quantify each metabolite with the right correction of the ME. As shown in Figure 5, for some metabolites, mainly those eluted before 3 min, external calibration curves are not acceptable. The ratio (peak area of endogenous compound/peak area of IS) corrected the calibration curves if IS undergoes the same ME as its related metabolite. The purchase of the labeled IS corresponding to each metabolite would be too expensive. The present approach, using 28 IS, proposed a cost-effective solution. The IS was selected to represent the metabolic pathways studied in order to be as close as possible to the metabolites of interest. To obtain as many labeled IS as metabolites, previous works reported the use of fully ^13^C metabolites produced using yeasts [3,6]. Although this approach is promising because it brings a huge number of labeled IS, it presents some limits. First, the quantity of IS added to samples is not known and the signals obtained for IS are not necessarily comparable to the signal of metabolites. It does not hinder the quantification process, but it does not correspond to optimal analytical work. Secondly, according to the yeast used to produce full ^13^C metabolites, some metabolites may be absent. For example, in previous work using ^13^C labeled extracts from *Pichia pastoris*, some metabolites exhibited concentrations too low to be used as IS [3]. This led the authors to use IS differently for the metabolites for some of them (i.e., ^13^C GMP for CMP, or ^13^C fumaric acid for oxaloacetic acid).

Considering the ME, for quantitative analysis, calibration curves have to be performed in extracts from the studied same cell line. As metabolites are endogenous compounds, it is not possible to spike them on “blank” cell extracts. The first option is to obtain a surrogate matrix of cell extracts [4,20]. However, as indicated above, the surrogate matrix does not mimic the real matrix. If a limited number of metabolites are studied, two approaches can be proposed. The first one is the use of standard addition calibration. However, it is time-consuming and needs large amounts of samples. The second one consists in using labeled metabolites as standards (surrogate analyte approach) by spiking cell extracts in order to obtain calibration curves [21]. The drawback is that it is expensive and IS with different labeling than the standards should be used.

It is known that quantities of metabolites produced by a cell line in different cell culture batches are different due to biological variability linked to several parameters (culture media composition, cell density, proliferation rate) [6,7,22]. Thus, is it relevant to consider an absolute quantification of each metabolite since it could not be compared to results from another experiment? A relative quantification approach can be proposed, making it possible to compare two situations (i.e., treated vs. non-treated cells, sensitive vs. resistant cells, etc.). In this case, the quantification can be carried out using a spiked non-biological matrix, such as water. Although the ME is not considered, the main drawback of this approach is that it is necessary to adapt calibration curves for each metabolite studied to the area of expected concentration. Another approach is based on the peak area ratio of a metabolite and its corresponding IS [23,24]. This method was selected in the present work.

The detailed study of the impact of the in vitro cellular matrix on the variability of the LC-HRMS method showed that this depends on:

- (i) the nature of the metabolite. For example, in MDA-MB-231 cell extracts, the CV of cysteine and creatinine (values between 7 to 19%) were higher than those of arginine and leucine (all CV ≤ 5%).
- (ii) for the same metabolite in a given cell line, the cell number. For some metabolites, such as carnitine or phenylalanine, the cell number has no impact on the CV (all values < 5%). For other metabolites, the cell number influenced the CV. For example, in HepaRG cells, for choline phosphate or hydroxyproline, the values of CV decreased with increasing cell number: 19% at 0.5 × 10^5^ cells and <10% for higher cell numbers for choline phosphate; 20% at 0.5 × 10^5^ cells, 12% at 1 × 10^5^ cells, and <10% for higher cell numbers for hydroxyproline. This indicates that it is better to perform analysis with a high cell number. Nevertheless, for some other metabolites, such as isocitric acid in MDA-MB-231 cells, the values of CV were lower (<15%) for 0.5, 1, 2, and 3 × 10^5^ cells than for 5 and 7 × 10^5^ cells (23% and 16%). Thus, the optimal cell number for analysis is not identical for each metabolite. Either a compromise has to be found or several injections corresponding to different numbers of cells should be performed.
- (iii) for the same metabolite and a given cell number, the nature of the cell line. For a low number of metabolites, such as aspartic acid, carnitine, glutamic acid, and phenylalanine, the variability was similar for MDA-MB-231 and HepaRG cell extracts. However, for the majority of metabolites, values of CV were different between MDA-MB-231 and HepaRG cells. For example, for arginine, CV values were lower than 4% for MDA-MB-231 cells, while they reached 18% for HepaRG cells. It was also observed for tryptophan with CV less than 10% for MDA-MB-231 cells and as high as 21% for HepaRG cells. If we focus on glutamine, we conclude that the optimal cell number was 0.5 × 10^5^ for MDA-MB-231 cells (CV = 3%) and 2 × 10^5^ for HepaRG cells (CV = 3%).

The impact of the nature of the cell line on the performance of the method was also shown with the study of intra-day precision. Five metabolites exhibited one or two CV values were higher than 20% in one cell line. While Serafimov et al. observed a better intra-day precision for metabolites from HeLa in vitro cell extracts detected after negative ESI than those after positive ESI [6], it seems not to be linked to the ionization mode in the present study. Indeed, it concerned two metabolites in negative mode (isocitric acid and succinic acid) and three metabolites in positive mode (spermidine, NAD, and tryptophan). Thus, we hypothesized that the cell type is the key parameter. Succinic acid, NAD, and tryptophan exhibited RT between 9.7 min and 14.7 min. However, we have shown a higher ME corresponding to ion suppression in HepaRG than in MDA-MB-231 cell extracts for internal standards with RT over 10 min (see succinic acid-D4, phenylalanine-^13^C9,^15^N, and tyrosine-^13^C9,^15^N in Figure 4). Thus, the higher variability observed for these metabolites in HepaRG cells may be due to metabolites not being detected in the present method but eluted around 10 min and present in higher quantities in HepaRG than in MDA-MB-231 cell extracts. Thus, the overall quantity of cellular metabolites present in the cell extract should influence the performance of the method. Nevertheless, this parameter is not known for each cell line and it is, therefore, difficult to anticipate the behavior of the analytical method for each cell line.

Overall, these results indicated that the performance of the method depend on the nature of the metabolite, the cell number, and the nature of the cell line, and this has consequences on the processing of experiments and the interpretation of the results.

For the processing of experiments, firstly, performing analysis in one analytical batch should be preferred since within-run variability is lower than the between-run (Figure 1). Thus, in the case of replicated experiments obtained from different cell culture batches, extracts have to be stored at −80 °C after cell lysis in order to perform analysis on all samples in the same run. Additionally, the cell number is a key parameter for in vitro cell extract metabolomic analysis. Our results indicated that the optimal cell number to obtain the lowest variability differs between metabolites. As shown in Figure 5, an increase in the cell number did not always result in an increase in signal. Thus, a high cell number is not the optimal condition for all metabolites and a compromise has to be found. It should be decided whether it is better to favor some metabolites of interest to the detriment of others or to define a cell number not optimal but suitable for a maximum of metabolites. The answer will depend on the applications. To help the researcher in the selection of the cell number, a PCA approach was proposed in the present work (Figure 7). It allows easy visualization of the cell number bringing the lowest variability.

Concerning the interpretation of the results, the level of significance to use to compare two samples or two conditions depends on the analytical performances. Thus, it differs again according to the nature of cells, the cell number, and the metabolite. For example, at 1 × 10^5^ cells (the cell number associated with the lowest variability in the present work), in HepaRG extracts, cysteine, fumaric acid, and serine exhibited CV values of 17%, 6%, and 4%, respectively. Thus, a difference of 15% in results between the two samples would be significant for fumaric acid and serine, but not for cysteine. Still, at 1 × 10^5^ cells, methionine showed CV values of 6% in MDA-MB-231 and 4% in HepaRG extracts, and pyroglutamic acid 5% in MDA-MB-231 and 14% in HepaRG extracts. Thus, a difference of 15% in results between the two samples would be significant for methionine in both cell lines, while for pyroglutamic acid, only in MDA-MB-231 extracts.

## 4. Materials and Methods

### 4.1. Chemicals and Reagents

Labeled and non-labeled metabolites came from Sigma-Aldrich (St-Quentin-Fallavier, France) or Eurisotop (Saint-Aubin, France). Methanol (MeOH) of HPLC-grade was obtained from Sigma-Aldrich and formic acid (FA) from Carlo Erba reagents (Val de Reuil, France). Water filtered with an Elga Purelab (Flex system, High Wycombe, UK) was used in all experiments.

### 4.2. Cell Culture and Sample Preparation

Human breast cells (MDA-MB-231) were from the American Type Culture Collection (ATCC) and cultivated in an RPMI medium (Gibco) supplemented with 10% fetal bovine serum (Thermofischer Scientific, Villebon sur Yvette, France), 2 mM L-glutamine, 100 U/mL penicillin, 100 mg/mL streptomycin, and 2 µg/mL fungizone at 37 °C with 5% CO_2_. Cells were tested for *Mycoplasma* every two weeks. For the preparation of metabolic extracts, cells were seeded in T75 flasks and incubated for 72 h with the replacement of media after 48 h. Cells were washed twice with cold PBS in the flask and 3 mL of cold MeOH/H_2_O/FA 68.5/28.5/3 was added. After 5 min of incubation on ice, the supernatant was recovered and aliquoted in tubes that were frozen for 20 s in liquid nitrogen and stored at −80 °C until analysis.

HepaRG cells, purchased from Biopredic International™ (Rennes, France), were grown at a low density in Williams’ E medium and were supplemented with 10% FCS, 100 units/mL penicillin, 100 µg/mL streptomycin, 5 µg/mL insulin, 2 mM glutamine, and 50 µM hydrocortisone hemisuccinate. After 2 weeks, the culture medium was supplemented with 1% DMSO, and the cells were left to differentiate for 1 week (confluent DMSO-treated cells). Cells were washed twice with cold PBS in a flask and 3 mL of cold MeOH/H_2_O/FA 68.5/28.5/3 was added. After 5 min incubation on ice, the supernatant was recovered and aliquoted in tubes that were frozen for 20 s in liquid nitrogen and stored at −80 °C until analysis.

For each cell line, an extract corresponding to 10^8^ cells was prepared. Thus, all experiments were performed on the same cell extract.

Prior to analysis, extracts were thawed to room temperature. Then, a mix of IS (Table S1) was added. After homogenization, samples were centrifuged. The supernatant was evaporated to dryness under nitrogen. The dry residue was resuspended in 200 µL mobile phase (0.1% of FA in water) and 10 µL was injected into the LC-HRMS system.

### 4.3. Determination of Protein Content

Total cellular proteins were extracted from pellets of 1, 2, and 5 million cells using a 20, 40, and 100 µL RIPA buffer, respectively. After adding the buffer and vortexing, tubes were incubated on ice for 1 h and centrifuged at high speed (15 min, 12,000× *g*, 4 °C). Protein concentrations of supernatants were determined with the Bradford method.

### 4.4. LC-HRMS Method

The LC-HRMS instrumentation included an Ultimate 3000 system (ThermoFisher Scientific^™^, Bremen, Germany) equipped with two ternary pumps and a Q-Exactive Plus Orbitrap mass spectrometer (ThermoFisher Scientific^™^, Bremen, Germany) equipped with a heated electrospray ionization (HESI) source. The chromatographic separation, adapted from Liu et al., was performed on a Synergi HydroRP column (250 × 2 mm; 4 µm, Phenomenex) [13]. The mobile phase was composed of 0.1% of FA in water (A) and 0.1% of FA in MeOH (B). The flow rate was 200 µL/min except for the re-equilibration step, during which it was set at 300 µL/min. The gradient elution was as follows: 0 min, 100% A; 2 min, 100% A, 20 min, 20% A; 22 min, 20% A; 22,1 min 100% A; and 37,1 100% A. The HESI source operated alternatively in negative and positive modes. The spray voltage was set at 2.5 kV in negative and positive modes. The pressure of nitrogen sheath gas and auxiliary gas was maintained at 25 and 10 units (arbitrary units), respectively. The capillary temperature was 350 °C. The mass spectrometer operated in full scan mode (60–900 *m*/*z*) with 70,000 for resolution, 250 ms for max injection time (IT), and 10^6^ for automatic gain control (AGC) target for both positive and negative ionization. The data-dependent MS^2^ (ddMS^2^) mode with 17,500 for resolution, 50 ms for max IT, and 10^5^ for AGC target was performed for the fragmentation of metabolites. This mode was applied on the 5 most abundant metabolites observed after each full scan (Top 5) for positive ionization and on the top 3 for negative ionization, with a dynamic exclusion of 8 s. The optimal collision energy employed was determined for each metabolite.

Two programs were used to build databases containing all information obtained from pure solutions of metabolites. TraceFinder^®^ 4.1 Forensic software (ThermoFisher Scientific™, Bremen, Germany) allowed the identification of metabolites according to RT, exact mass and isotopic pattern of the molecular ion, and exact mass of fragments. TraceFinder^®^ 4.1 Forensic software was linked to mzVault^®^ 1.0sp1 software (ThermoFisher Scientific™, Bremen, Germany) which contained the fragmentation spectrum of each metabolite for defined collision energy. These data have been implemented by the analysis of pure standards. A total of 84 metabolites were implemented in the LC-HRMS processing method (Table S1).

### 4.5. Study of the Performances of the Method on In Vitro Cell Extracts

The study of the impact of cellular matrix on the performances of the method was performed on the same cell extract for each cell line and following the workflow presented in Figure 2.

The variability of RT for each metabolite was evaluated by the analysis of 30 cellular extracts of MDA-MB-231 and HepaRG cell lines. Carryover was estimated by the analysis of a mobile phase after the analysis of cell extracts corresponding to 7 × 10^5^ cells. This parameter was verified three times. The ME was evaluated using labeled metabolites spiked in MDA-MB-231 cell extracts corresponding to 1 × 10^5^ and 5 × 10^5^ cells. The area of each labeled metabolite spiked in a cell extract was compared with the area of the same quantity of labeled metabolites spiked in water. The ME was expressed as [100—(peak area in cell extract × 100)/peak area in water]. To complete the study of the ME, the influence of the nature of cell lines on signal intensities was studied by spiking cell extracts from 3 × 10^5^ MDA-MB-231 cells and HepaRG cells with the same amount of IS. Results were expressed as the peak area of each internal standard in HepaRG cells compared with the peak area in MDA-MB-231 cells. Linearity of the method for each metabolite was assessed by analyzing cell extracts from MDA-MB-231 and HepaRG cell lines corresponding to increased numbers of cells: 0.5 × 10^5^, 1 × 10^5^, 2 × 10^5^, 3 × 10^5^, 5 × 10^5^, and 7 × 10^5^. The results of the linearity were expressed as the bias between the calculated cell number and theoretical cell number for each level. Within-run variability was determined by the analysis of 5 calibration curves on the same run in one day. Between-run variability was estimated by the analysis of 15 calibration curves on 3 runs performed on 3 days. Results of within-run and between-run variabilities were expressed as coefficients of variation for each level.

### 4.6. Principal Component Analysis

The PCA was computed using the dudi.pca function of the ade4 R package with the following parameters: center = TRUE and scale = TRUE.

## 5. Conclusions

In the present work, we have demonstrated the impact of the nature of the cell line and the cell number on the matrix effect and the variability of an LC-HRMS metabolomic method. We have also shown that this impact differs depending on the metabolite. The key parameters to determine before performing analysis on real samples are the optimal cell number and the variability of the method for each metabolite. Experiments 2 and 3 presented in Figure 2 show all data required to master the key parameters.

## Data Availability

The data that support the findings of this study are available from the corresponding author upon reasonable request.

## Supplementary Materials

The following supporting information can be downloaded at https://www.mdpi.com/article/10.3390/ijms24043770/s1.
