# Supplementary material for: A Proposed Methodology to Deal with the Impact of In Vitro Cellular Matrix on the Analytical Performances of a Targeted Metabolomic LC-HRMS Method
Source: Int J Mol Sci. 2023 Feb 13;24(4):3770. doi: 10.3390/ijms24043770 (PMC9965333; doi:10.3390/ijms24043770)
Supplement: Supplementary file 1 [file ijms-24-03770-s001.zip › ijms-2146364-supplementary.pdf]

Table S1. List of the 88 metabolites included in the LC-MS/HRMS method and their related internal standard. Positive and negative ionization modes corresponded to $[M+H]^+$ and $[M-H]^-$ ions, respectively were used for the quantification.

Metabolite	Retention time (min)	Ionization mode	m/z	Internal standard
Acetylcarnitine	5,1	Positive	204.12303	Acetylcarnitine (D3)
Acetylcholine	3,5	Positive	146.11756	Proline (13C5, 15N)
Aconitic acid	10,0	Positive	175.02371	Citric acid (D4)
Adenine	6,6	Positive	136.06177	Methionine (13C5, 15N)
Adenosine	11,1	Positive	268.10403	ThioNAD
Alanine	2,7	Positive	90.05500	Alanine (13C3, 15N)
Alpha-ketoglutarate	5,6	Negative	145.01425	Succinic acid (D4)
AMP	9,0	Positive	348.07036	ThioNAD
Arginine	2,8	Positive	175.11900	Arginine (13C6, 15N4)
Asparagine	2,7	Positive	133.06080	Serine (13C3, 15N)
Aspartic acid	2,7	Positive	134.04478	Aspartic acid (13C4, 15N)
Biotine	17,6	Negative	243.08089	Phenylalanine (13C9, 15N)
Carnitine	3,1	Positive	162.11247	MethylCarnitine (D3)
Choline	3,3	Positive	104.10699	Choline (D9)
Choline phosphate	2,9	Positive	184.07332	Choline (D9)
Citric acid	8,4	Negative	191.01973	Citric acid (D4)
Citrulline	3,0	Positive	176.10297	Citrulline (D4)
CMP	4,8	Positive	324.05913	ThioNAD
Coumaric acid	19,4	Positive	165.05462	Citric acid (D4)
Creatine	3,4	Positive	132.07675	Proline (13C5, 15N)
Creatinine	3,1	Positive	114.06619	Proline (13C5, 15N)
Cysteine	3,0	Positive	122.02700	Citrulline (D4)
Fructose-1,6-bisphosphate	2,8	Negative	338.98877	Fructose-6-Phosphate (13C6)
Fumaric acid	9,8	Negative	115.00370	Succinic acid (D4)
GABA	2,9	Negative	102.05605	Glutamic acid (13C5, 15N)
GDP mannose	9,8	Negative	604.06990	Fructose-6-Phosphate (13C6)
Glutamic acid	2,9	Positive	148.06040	Glutamic acid (13C5, 15N)
Glutamine	2,8	Positive	147.07640	Aspartic acid (13C4, 15N)
Glycero-3-phosphocholine	2,9	Positive	258.11010	Choline (D9)
Glycine	2,7	Positive	76.03930	Glycine (13C2, 15N)
GMP	9,8	Positive	364.06528	ThioNAD
Hexanoylcarnitine	7,1	Positive	260.18563	Acetylcarnitine (D3)
Hexose phosphate	2,8	Negative	259.02244	Fructose-6-Phosphate (13C6)
Histidine	2,7	Positive	156.07675	Histidine (13C6, 15N3)
Hydroxyindolacetic acid	11,9	Positive	192.06552	Phenylalanine (13C9, 15N)
Hydroxyproline	8,7	Positive	132.06552	Threonine (13C4, 15N)
Hypoxanthine	8,2	Positive	137.04579	ThioNAD
Inosine	11,6	Negative	267.07349	ThioNAD
Isocitric acid	4,7	Negative	191.01973	Citric acid (D4)

Isoleucine	9,7	Positive	132.10190	Isoleucine (13C6. 15N)
Lactic acid	5,6	Negative	89.02442	Succinic acid (D4)
Leucine	10,1	Positive	132.10190	Leucine (13C6)
Lysine	2,3	Positive	147.11280	Lysine (13C6, 15N2)
Malic acid	4,5	Negative	133.01425	Succinic acid (D4)
Methionine	5,6	Positive	150.05830	Methionine (13C5, 15N)
Methylhippuric acid	18,0	Negative	192.06662	Phenylalanine (13C9, 15N)
N-acetylglucosamine phosphate	3,0	Negative	227.03261	Fructose-6-Phosphate (13C6)
N-acetylneuraminic acid	3,4	Negative	308.09870	Citrulline (D4)
N-acetylornithine	3,9	Positive	175.10772	Ornithine (13C5)
N-acetylputrescine	3,8	Positive	131.11789	Spermine (D8)
N-acetylserine	2,9	Positive	148.06043	Serine (13C3, 15N)
N-acetylspermidine	2,8	Positive	188.17570	Fructose-6-Phosphate (13C6)
N-acetylspermine	2,5	Positive	245.23359	Spermine (D8)
NAD	10,0	Positive	664.11639	ThioNAD
NAM	9,7	Positive	123.05528	NAM (13C6)
Nicotinamide-N-oxide	7,0	Positive	139.05020	NAM (13C6)
N-methylnicotinamide	3,1	Positive	137.07094	NAM (13C6)
NMN	4,8	Positive	335.06388	NAM (13C6)
Norleucine	9,3	Positive	132.10190	Leucine (13C6)
O-propanoylcarnitine	10,1	Positive	218.13868	Tyrosine (13C9, 15N)
Oxidized glutathione	10,2	Positive	613.15920	Tyrosine (13C9, 15N)
Ornithine	2,4	Positive	133.09715	Ornithine (13C5)
Oxaloacetate	8,7	Negative	130.99860	Citric acid (D4)
Oxoglutaric acid	8,5	Positive	147.02880	Citric acid (D4)
Pantothenic acid	13,6	Positive	220.11795	Phenylalanine (13C9, 15N)
Phenylalanine	12,5	Positive	166.08626	Phenylalanine (13C9, 15N)
3-phosphoglyceric acid	3,2	Negative	184.98566	Proline (13C5, 15N)
Proline	3,3	Positive	116.07060	Proline (13C5, 15N)
Putrescine	2,3	Positive	89.10732	Lysine (13C6, 15N2)
Pyroglutamic acid	8,8	Positive	130.04987	Glutamic acid (13C5, 15N)
Pyruvate	4,4	Negative	87.00877	Citric acid (D4)
Reduced glutathione	6,7	Negative	306.07650	Methionine (13C5, 15N)
Ribose phosphate	3,0	Negative	229.01190	Fructose-6-Phosphate (13C6)
SAH	10,4	Positive	385.12890	ThioNAD
SAM	2,9	Positive	399.14450	Citrulline (D4)
Serine	2,7	Positive	106.04990	Serine (13C3, 15N)
Spermidine	2,1	Positive	146.16517	Spermine (D8)
Spermine	2,0	Positive	203.22300	Spermine (D8)
Succinic acid	9,7	Negative	117.01930	Succinic acid (D4)
Thiamine	3,5	Positive	265.11176	Citrulline (D4)
Threonine	2,9	Positive	120.06552	Threonine (13C4, 15N)
Tryptophane	14,7	Positive	205.09715	Phenylalanine (13C9, 15N)
Tyrosine	10,1	Positive	182.08117	Tyrosine (13C9, 15N)

UDP N-Acetylhexosamine	5,0	Positive	608.08885	Valine (13C5, 15N)
UDP hexose	8,5	Negative	565.04770	Valine (13C5, 15N)
UMP	6,3	Positive	325.04314	ThioNAD
Valine	4,4	Positive	118.08626	Valine (13C5, 15N)
Xanthine	10,5	Positive	153.04070	Tyrosine (13C9, 15N)
